# From the Cochrane Library: Optical Coherence Tomography for Diagnosing Skin Cancer in Adults

**DOI:** 10.2196/41355

**Published:** 2023-03-13

**Authors:** Colin Burnette, Torunn E Sivesind, Robert Dellavalle

**Affiliations:** 1 Nova Southeastern University College of Osteopathic Medicine Davie, FL United States; 2 Department of Dermatology University of Colorado Anschutz Medical Campus Aurora, CO United States; 3 Rocky Mountain Reginal Veterans Affairs Medical Center Aurora, CO United States

**Keywords:** systematic review, optical coherence tomography, tomography, diagnostic imaging, optical imaging, laser, skin lesions, diagnostic techniques, melanoma, basal cell carcinoma, cancer, skin cancer, clinical, cell, diagnose

Since the development of optical coherence tomography (OCT), technological improvements have made this diagnostic imaging tool adaptable to clinical settings [1]. OCT provides an alternative modality for assessing skin abnormalities. The standard procedure for the evaluation of suspicious skin lesions involves visual inspection, often with dermoscopy, which may be followed by a biopsy for histological confirmation of diagnosis. OCT magnifies the skin for a more detailed examination compared to standard techniques. Using near-infrared light, tissue can be viewed on a microscale in real time [1]. Although the use of OCT is more prevalent, its effectiveness in diagnosing skin cancer and its overall accuracy are insufficiently characterized [2].

A 2018 Cochrane review, “Optical Coherence Tomography for Diagnosing Skin Cancer in Adults,” provides an in-depth review of the accuracy of OCT for detecting skin abnormalities. Data from five test accuracy studies were obtained, permitting comparison of the index test to a reference standard. The diagnostic accuracy of OCT was assessed for melanoma (n=2) and keratinocyte carcinomas (n=3). There were insufficient data to determine the diagnostic accuracy of OCT for melanoma or cutaneous squamous cell carcinoma. In a sample of 346 lesions (n=2 studies), the sensitivity and specificity of OCT for detecting basal cell carcinoma (BCC) was potentially superior versus visual assessment and dermoscopic exam—however, given a limited sample size and the high prior probability of BCC, results must be interpreted cautiously. Applied to a hypothetical population (n=1000 people), OCT had better outcomes than visual and dermoscopic exams, correctly identifying 53 more BCC lesions while reducing the incidence of false positives and unnecessary excisions by 87 [2].

The high sensitivity and specificity of OCT for BCC diagnosis are noted in subsequent reviews and expert consensus [3,4]. An increasing emphasis on aesthetically mindful outcomes highlights the importance of noninvasive modalities like OCT, with minimally invasive treatment options such as Mohs surgery increasing over 300% in a 15-year span [5]. For BCC, the use of OCT could potentially reduce the need for biopsy and histological confirmation by as much as 33% [3]. Despite its promise for BCC diagnosis, OCT accuracy may not be generalized to all suspect lesions. In cases of nonmelanoma lesions where the differential excludes BCC, OCT may result in overdiagnosis and, in some reports, a high incidence of false negatives [4]. Applying OCT to other skin lesions may, therefore, result in negative consequences (eg, misdiagnosis, repeat testing); with insufficient data regarding its accuracy [2], this raises the question of whether the benefit of noninvasive testing outweighs the risks.

The insufficient number of studies available for inclusion, study heterogeneity, and restricted study populations limit the conclusions that may be drawn regarding the diagnostic utility of OCT [2]. Included studies exhibited poor reporting, prohibiting a risk of bias assessment; additionally, study results may not be applicable to standard clinical practice [2]. Future studies should be of high methodological quality, clarify recruitment methods, and incorporate a blinded reference standard for results comparison [2]. Considering rapid technological advancements in OCT [1], well-conducted studies may enable its broader application to clinical practice in the near future.

## Abbreviations

BCC: basal cell carcinoma
OCT: optical coherence tomography

## References

[1] Zysk AM, Nguyen FT, Oldenburg AL, Marks DL, Boppart SA (2007). Optical coherence tomography: a review of clinical development from bench to bedside. J Biomed Opt.

[2] Ferrante di Ruffano L, Dinnes J, Deeks JJ, Chuchu N, Bayliss SE, Davenport C, Takwoingi Y, Godfrey K, O'Sullivan C, Matin RN, Tehrani H, Williams HC, Cochrane Skin Cancer Diagnostic Test Accuracy Group (2018). Optical coherence tomography for diagnosing skin cancer in adults. Cochrane Database Syst Rev.

[3] Dorrell DN, Strowd LC (2019). Skin cancer detection technology. Dermatol Clin.

[4] Pathania YS, Apalla Z, Salerni G, Patil A, Grabbe S, Goldust M (2022). Non-invasive diagnostic techniques in pigmentary skin disorders and skin cancer. J Cosmet Dermatol.

[5] Wang DM, Morgan FC, Besaw RJ, Schmults CD (2018). An ecological study of skin biopsies and skin cancer treatment procedures in the United States Medicare population, 2000 to 2015. J Am Acad Dermatol.

